# Neuropathological hallmarks in the post-mortem retina of neurodegenerative diseases

**DOI:** 10.1007/s00401-024-02769-z

**Published:** 2024-08-19

**Authors:** Frederique J. Hart de Ruyter, Manon J. A. P. Evers, Tjado H. J. Morrema, Anke A. Dijkstra, Jurre den Haan, Jos W. R. Twisk, Johannes F. de Boer, Philip Scheltens, Femke H. Bouwman, Frank D. Verbraak, Annemieke J. Rozemuller, Jeroen J. M. Hoozemans

**Affiliations:** 1 Amsterdam UMC, Vrije Universiteit Amsterdam, Pathology, Amsterdam Neuroscience, De Boelelaan 1117, 1081 HV Amsterdam, The Netherlands; 2 Amsterdam UMC, Vrije Universiteit Amsterdam, Alzheimer Center Amsterdam, Neurology, De Boelelaan 1117, Amsterdam, The Netherlands; 3 Epidemiology and Data Science, Vrije Universiteit Amsterdam, De Boelelaan 1117, Amsterdam, The Netherlands; 4 LaserLaB, Physics and Astronomy, Vrije Universiteit Amsterdam, De Boelelaan 1081, Amsterdam, The Netherlands; 5 Amsterdam UMC, Vrije Universiteit Amsterdam, Ophthalmology, De Boelelaan 1117, Amsterdam, The Netherlands

**Keywords:** Immunohistochemistry, Neurodegeneration, Eye, retina, Neuropathology, Amyloid beta, Tau, Alpha-synuclein, TDP-43

## Abstract

The retina is increasingly recognised as a potential source of biomarkers for neurodegenerative diseases. Hallmark protein aggregates in the retinal neuronal tissue could be imaged through light non-invasively. Post-mortem studies have already shown the presence of specific hallmark proteins in Alzheimer’s disease, primary tauopathies, synucleinopathies and frontotemporal lobar degeneration. This study aims to assess proteinopathy in a post-mortem cohort with different neurodegenerative diseases and assess the presence of the primary pathology in the retina. Post-mortem eyes were collected in collaboration with the Netherlands Brain Bank from donors with Alzheimer’s disease (*n* = 17), primary tauopathies (*n* = 8), synucleinopathies (*n* = 27), frontotemporal lobar degeneration (*n* = 8), mixed pathology (*n* = 11), other neurodegenerative diseases (*n* = 6), and cognitively normal controls (*n* = 25). Multiple cross sections of the retina and optic nerve tissue were immunostained using antibodies against pTau Ser202/Thr205 (AT8), amyloid-beta (4G8), alpha-synuclein (LB509), pTDP-43 Ser409/410 and p62-lck ligand (p62) and were assessed for the presence of aggregates and inclusions. pTau pathology was observed as a diffuse signal in Alzheimer’s disease, primary tauopathies and controls with Alzheimer’s disease neuropathological changes. Amyloid-beta was observed in the vessel wall and as cytoplasmic granular deposits in all groups. Alpha-synuclein pathology was observed as Lewy neurites in the retina in synucleinopathies associated with Lewy pathology and as oligodendroglial cytoplasmic inclusions in the optic nerve in multiple system atrophy. Anti-pTDP-43 generally showed typical neuronal cytoplasmic inclusion bodies in cases with frontotemporal lobar degeneration with TDP-43 and also in cases with later stages of limbic-associated TDP-43 encephalopathy. P62 showed inclusion bodies similar to those seen with anti-pTDP-43. Furthermore, pTau and alpha-synuclein pathology were significantly associated with increasing Braak stages for neurofibrillary tangles and Lewy bodies, respectively. Mixed pathology cases in this cohort consisted of cases (*n* = 6) with high Braak LB stages (> 4) and low or moderate AD pathology, high AD pathology (*n* = 1, Braak NFT 6, Thal phase 5) with moderate LB pathology, or a combination of low/moderate scores for different pathology scores in the brain (*n* = 4). There were no cases with advanced co-pathologies. In seven cases with Braak LB ≥ 4, LB pathology was observed in the retina, while tau pathology in the retina in the mixed pathology group (*n* = 11) could not be observed. From this study, we conclude that the retina reflects the presence of the major hallmark proteins associated with neurodegenerative diseases. Although low or moderate levels of copathology were found in the brains of most cases, the retina primarily manifested protein aggregates associated with the main neurodegenerative disease. These findings indicate that with appropriate retinal imaging techniques, retinal biomarkers have the potential to become highly accurate indicators for diagnosing the major neurodegenerative diseases of the brain.

**Supplementary Information:**

The online version contains supplementary material available at 10.1007/s00401-024-02769-z.

## Introduction

With the increasing number of patients suffering from dementia [45], there is an urgent need for the development of minimal-invasive, low-cost biomarkers suitable for (pre)clinical screening of the population. As part of the central nervous system, the retina seems subject to the same neuropathological processes that affect the brain. Recent post-mortem evidence suggests that the retina contains the same pathological proteins as found in the brains of cases with neurodegenerative diseases [17, 21, 46, 47, 49, 54]. Located outside the cranium, the retina is the only place where we can directly visualise neuronal tissue utilising non-invasive imaging techniques through light [16, 18]. Therefore, retinal biomarkers may be promising for diagnosing brain-related neurodegenerative diseases.

Most neurodegenerative diseases are characterised by the pathologic accumulation of proteins associated with neuronal dysfunction and neurodegeneration [53]. The diagnosis of protein-based neurodegenerative diseases relies on a specific clinical phenotype and a distinct pattern of protein aggregates. In recent years, studies have confirmed the importance of multi-pathology for the clinical phenotype and disease course [15, 26, 32, 48, 52]. Although not fully elucidated, neuropathological comorbidity has been associated with specific brain regions [25], the age of onset [48], the severity of the disease [19, 25], and with promoting cognitive decline in Alzheimer's disease (AD) [42] and synucleinopathies [19, 44]. Neuropathological studies show that only 35–50% of the cases with AD have pure tau and amyloid-beta (Aβ) pathology [32, 52]. Additional mixed pathologies are seen mostly with alpha-synuclein (αSyn) (41–55%) reflecting limbic amygdala-only pathology [58] and cortical pathology in the form of Parkinson’s disease (PD) and dementia with Lewy bodies (DLB), and TDP-43 pathology (33–40%) as part of limbic-predominant age-related TDP-43 encephalopathy neuropathologic changes (LATE-NC) [41, 43]. AD neuropathologic changes (ADNC) also occur without clinical AD and are then considered incidental or to be associated with a prodromal stage of AD [36]. In addition, the presence of incidental proteinopathies without an apparent clinical phenotype is acknowledged to occur with advancing age [27, 32, 51]. The diagnosis of the primary neurodegenerative disease responsible for the symptomatology is challenging due to the high prevalence of concomitant pathology.

How the retina reflects proteinopathy in cases with neuropathological comorbidity has not been elucidated. Most previous research on the post-mortem human retina has primarily focused on specific disease groups and individual proteins. These studies have shown that hallmark pathological features present in the brain tissue of primary neurodegenerative diseases can also be observed in the retina [17, 21, 46, 54]. Diffuse phosphorylated tau (pTau) was observed in AD [17], primary tauopathies [21, 54], and control cases with early Braak stages for neurofibrillary tangles [21]. α-Syn pathology was observed in the form of Lewy neurites and possibly Lewy bodies in the retina of donors with DLB [20, 46] and glial cytoplasmic inclusions in the optic nerve of donors with multiple system atrophy [20]. Finally, phosphorylated transactive response DNA-binding protein 43 (pTDP-43) inclusions were observed in the retinas of donors with frontotemporal lobar disease with TDP-43 (FTLD-TDP) [11], amyotrophic lateral sclerosis (ALS) [47] and chronic traumatic encephalopathy (CTE) [49]. Currently, most controversy exists on retinal Aβ deposits and their association with AD. One group has conducted a series of extensive experiments that indicate a correlation between the presence of intra- and extracellular Aβ deposits in AD patients and cognitive decline [13, 29, 30, 55]. However, other groups have failed to replicate these findings [17, 22, 54, 61], leading to further questions and debates about the role of retinal Aβ in this disease. Although a few studies have previously investigated cohorts, including multiple neurodegenerative diseases [21, 54], no study has looked at the role of concomitant pathology on the retina. This lack of exploration has led to a gap in knowledge, making it unclear if pathologic protein deposits in the retina are exclusive to the main neurodegenerative disease or if they are also present in the retina of donors with concomitant pathology in the brain.

If the retina were to be utilised for biomarker development, the predictive value of retinal biomarkers for brain proteinopathy should be assessed and the presence of co-pathology needs to be explored [5]. The present work extends the research on the retina [11, 17, 20, 21], determining the retinal manifestation of the four major protein aggregates (pTau, Aβ, αSyn and pTDP-43) associated with neurodegenerative diseases. Furthermore, this study aims to assess whether the retina mirrors proteinopathy comorbidity similar to what is observed in the brain and whether retinal biomarkers could be applied to detect the primary neurodegenerative disease. Elucidating the above would increase our understanding of the significance of the retina in reflecting the presence of brain neuropathology.

## Methods

### Post-mortem tissue

Post-mortem eyes and brain tissue of 102 donors were collected from 2009 until 2022 by the Netherlands Brain Bank (Amsterdam, The Netherlands, www.brainbank.nl), according to a donor program approved by the VUmc medical ethics committee. All donors consented to the use of tissue and clinical records for research purposes in compliance with ethical standards. Brain autopsies were performed according to the Code of Conduct of Brain Net Europe [28].

Brain autopsy was performed within 12 h post-mortem, after which brain tissue was formalin-fixed (10%; 4 weeks) and embedded in paraffin. For this study, multiple brain regions were assessed as part of the protocol of the Netherlands Brain Bank. Neuropathological diagnosis was performed (AR) according to the guidelines of the BrainNet Europe Consortium [1, 4], including assessment of Alzheimer’s disease neuropathological changes (ADNC) [39], i.e. Thal phase for Aβ [22, 57], Braak stage for NFTs (Braak NFT stage) [7], CERAD score for neuritic plaque pathology [37], and Braak stage for Lewy bodies (Braak LB stage) [8]. Cases were grouped according to clinicopathological diagnosis in typical Alzheimer’s disease (*n* = 13), atypical Alzheimer’s disease [40] (*n* = 4), a “mixed pathology” group existing of cases with more than one neurodegenerative disease in an advanced stage (i.e. Braak NFT stage > 3, Thal amyloid phase > 2, Braak LB stage > 3, severe ARTAG) (*n* = 11), primary tauopathies [9, 10, 14, 31] (*n* = 8), synucleinopathies [23, 24, 34] (*n* = 27), FTLD with underlying aggregation of TDP-43 [35] (*n* = 8), and other neurodegenerative diseases (*n* = 6) among which FTLD-FUS, multiple sclerosis, hydrocephalus adultorum, amyotrophic lateral sclerosis, vascular dementia and neuronal intranuclear inclusion disease, and controls. Clinically normal control cases without cognitive impairment were stratified into two groups based on neuropathology: controls with neuropathological changes (controls + , *n* = 18) (i.e. ADNC [36], incidental Lewy body disease [20], and other ageing-related neuropathological changes) and controls without neuropathological changes (controls-, *n* = 7). Neuropathological changes included ADNC, ageing-related tau astrogliopathy (ARTAG) [31], primary age-related tauopathy (PART) [14], LATE-neuropathologic changes (LATE-NC) [43], and cerebral amyloid angiopathy (CAA). Clinical records showed 13 cases with a medical history of ophthalmologic disease, including glaucoma (*n* = 6), age-related macular degeneration (*n* = 3), optic neuritis (*n* = 1), retinal detachment (*n* = 2) and macular pucker (*n* = 1). A history of ophthalmologic disease was not an exclusion criterion in this study. Cohort characteristics are shown in Supplementary Table 1. Of this cohort results have been reported earlier on 62 cases [21].

### Tissue preparation

Post-mortem enucleated eyes were fixed in 4% paraformaldehyde (PFA) for 48 h. The eye was dissected through the horizontal and vertical axis, resulting in temporal-superior, temporal-inferior, nasal-superior and nasal-inferior quadrants as previously described [17, 21]. Before embedding, the quadrants were dehydrated using the following protocol: 3 h in 4% PFA at 35 °C, 1 h in 70% ethanol at 35 °C, 1 h in 80% ethanol at 35 °C, 1 h in 96% ethanol at 35 °C, three times for 1 h in 100% alcohol at 35 °C, three times for 1 h in xylene at 35 °C, and four times for 1 h in paraffin at 62 °C.

### Immunohistochemistry

Tissue sections from formalin-fixed, paraffin-embedded retina at 10 µm thickness of the superior and inferior axis were sequentially mounted on TOMO glass slides (Matsunami, Osaka, Japan). Brain tissue at 7 µm thickness was mounted on Superfrost plus glass slides (VWR, Pennsylvania, USA). Slides were air-dried overnight at 37 °C before staining. Sections were deparaffinized and rehydrated using sequential incubations in xylene, alcohol and water. Endogenous peroxidase activity was suppressed by incubating the slides with 0.3% H_2_O_2_ in phosphate-buffered saline (PBS) pH 7.4 for 30 min at RT. Antigen retrieval was performed in 10 mM citrate buffer (pH 6.0) and heated using an autoclave (20 min at 121 °C). Sections were incubated with primary antibodies against pTau Ser202/Thr205 (AT8, Thermo Scientific, MA, USA, 1:800), αSyn (LB509, Thermo Scientific, MA, USA, 1:500), pTDP-43 Ser409/410 (Cosmo Bio USA, CA, USA, 1:6000), and p62-lck ligand_257-437_ (BD Biosciences, CA, USA, 1:2000), diluted in antibody diluent (Sigma-Aldrich, Saint Louis, USA) overnight at RT. For the detection of Aβ, the antibody 4G8 (BioLegend, San Diego CA, 1:1000) was used. The 4G8 antibody is widely applied in diagnostic neuropathology labs for the detection of Aβ [4]. In a previous study, various antibodies utilized for the detection of Aβ have been assessed on the retina. Findings from this study show that the 4G8 antibody demonstrates minimal overlap with the detection of APP when compared to a specific antibody for APP [17].

Sections immunostained with LB509 were additionally pre-treated with 100% formic acid (10 min at RT) and washed with PBS. The omission of the primary antibody was used as a negative control. After primary antibody incubation, the sections were washed with PBS, incubated with anti-mouse/rabbit HRP Envision (DAKO, Glostrup, Denmark) (30 min) and subsequently washed using PBS. 3,3’-Diaminobenzine (DAB; DAKO) and Liquid Permanent Red (LPR) were used as a chromogen for colour development (5 min at RT). Sections were counterstained with hematoxylin or anti-SOX-10 (Cell Marque, 1:100, clone EP268), dehydrated using alcohol and xylene and mounted using Quick-D (Klinipath; Duiven, The Netherlands).

### Immunoreactivity assessment and quantification

The presence and localisation of retinal pTau, Aβ, αSyn, pTDP-43 and p62 were assessed in 102 donors in the superior and inferior retina. For 28 donors, also optic nerve tissue was available, which was separately evaluated for αSyn pathology. Immunostained cross-sections were imaged using an Olympus VS200 slide scanner. The presence and localisation of pathology were assessed in 102 donors. Per case and immunostaining, at least two sections were immunostained; one section of the superior retina and one section of the inferior retina. Immunoreactivity for pTau was assessed following the staging for primary retinal tauopathy (PReT) [60]. Inclusions immunoreactive for αSyn, 4G8, pTDP-43 and p62 were scored as follows: few for 1–5 inclusions, moderate for 6–20 inclusions, many for more than 20 inclusions along the entire cross-section of the retina (from far peripheral to central retina). Retinal tissue was scored based on dichotomised scoring; a section was scored positive ( +) for the presence of pTau, αSyn positive inclusions, 4G8 positivity, pTDP-43 inclusions and ubiquitin-binding protein p62 inclusions as a marker for neuronal and glial inclusions[50], localised in the retina and/or optic nerve (Table 2). In brain tissue, the presence of pTau, αSyn, pTDP-43 and Aβ immunoreactivity was scored with an Olympus BX41 microscope in different brain regions with total surface area ranging from 2 to 4 cm^2^ per section [11, 20, 21]. Assessment of pathology in the brain and the retina was performed separately and masked for clinical diagnosis. Figures were composed with R (R Core Team, 2023, RRID:SCR_001905), GraphPad Prism (RRID:SCR_002798, version 9.3.1), Google Looker Studio (RRID:SCR_023549) and Adobe Photoshop (Adobe Systems Incorporated, RRID:SCR_014199, version 23.5).

### Statistical analysis

Demographic group differences based on neuropathological diagnosis were studied using Fisher’s exact test and one-way ANOVA. Binary and penalized likelihood logistic regression analysis were used to study the association between retinal pathology and neuropathological groups as well as brain pathology staging systems. All regression analyses were corrected for age at death and sex and were performed using SPSS Statistics (IBM SPSS Statistics, RRID: SCR_016479) version 29. A *P* value of < 0.05 was considered significant. To rule out an effect of ophthalmologic pathology on protein presence in the retina, all analyses were additionally performed after exclusion of the cases with posterior segment pathology. The exclusion of these cases did not change the statistical outcome.

### Data availability

All data generated or analysed during this study are included or referred to in this published article and its supplementary information files.

## Results

### Cohort description

Controls without pathology were significantly younger than the other groups (*P* < 0.001), and cases within the FTLD-TDP group were significantly younger than cases in the mixed pathology group (*P* = 0.02) (Table 1). There were no significant differences observed between the groups for sex (*P* = 0.07), disease duration (*P* = 0.16) or post-mortem interval (*P* = 0.11). The Alzheimer’s disease group showed a significantly higher Braak NFT stage (*P* < 0.001) and Thal amyloid phase (*P* < 0.001) compared to all other groups, as the synucleinopathies group had a significantly higher Braak LB stage (*P* < 0.001) than all other groups except for the mixed pathology group. Synucleinopathies additionally showed a significantly higher Braak NFT stage than both control groups. Detailed individual case information is provided in Supplementary Table 1.

**Table 1:** Group demographics in this study

	Controls −	Controls +	Typical Alzheimer’s disease	Atypical Alzheimer’s disease	Mixed pathology	Primary tauopathies	Synucleinopathy	FTLD-TDP	Other neurodegenerative diseases
	No pathology	Minimal pathology		PCA, EOAD	AD/LBD, LBD/PSP, ARTAG/AD	CBD, FTLD-tau, PSP, PART/ARTAG	PD, PDD, DLB, MSA	Type A, B, C, E	ALS, FTLD-FUS, Fragile X syndrome, hydrocephalus, MS, vascular dementia
	*n* = 7	*n* = 18	*n* = 13	*n* = 4	*n* = 11	*n* = 8	*n* = 27	*n* = 8	*n* = 6
Female, *n*	5	13	8	4	6	3	10	3	1
Age at death Mean ± SD	53 ± 12	80 ± 11	79 ± 9	69 ± 7	86 ± 9	72 ± 11	74 ± 8	66 ± 9	68 ± 14
Disease duration Median; min–max	n/a	n/a	90 (12–312)	108 (48–120)	96 (24–300)	114 (36–180)	132 (36–324)	48 (24–84)	120 (72–180)
Braak NFT stage Median; min–max	0	2 (1–3)	5 (4–6)	6 (4–6)	4 (1–6)	2 (1–3)	2 (0–3)	1 (0–2)	2 (0–2)
Thal amyloid phase Median; min–max	0	1 (0–4)	5 (3–5)	5	4 (0–5)	2 (0–3)	1 (0–3)	1 (0–3)	1 (0–2)
Braak LB stage Median; min–max	0	0 (0–4)	1 (0–4)	0	5 (0–6)	0	6 (3–6)	0	0
Cognitive state Median; min–max	0	0 (0–1)	2 (1–3)	3 (1–3)	2 (0–3)	1 (0–3)	0 (0–3)	3 (1–3)	2 (0–3)
Post-mortem interval eyes Median; min–max	9 (5–30)	8 (3–22)	7 (5–16)	7 (5–8)	7 (4–11)	6 (4–10)	6 (4–9)	6 (4–18)	7 (7–10)

Data are shown mean ± SD or median (minimum–maximum). Age at death is shown in years, disease duration in months and post-mortem interval in hours. The cognitive state is scored according to the clinical dementia rating (CDR) scale.

*ARTAG* age-related tau astrogliopathy; *CBD* corticobasal degeneration; *ALS* amyotrophic lateral sclerosis; *EOAD* early-onset Alzheimer’s disease; *FTLD* frontotemporal lobar degeneration; *DLB* dementia with Lewy bodies; *MS* multiple sclerosis; *MSA* multiple system atrophy; *n/a* not applicable; *PCA* posterior cortical atrophy; *PD(D)* Parkinson’s disease (with dementia); *PSP* progressive supranuclear palsy; *PART* primary age-related tauopathy

### Morphological appearance of proteinopathy in the retina

**Fig. 1:**
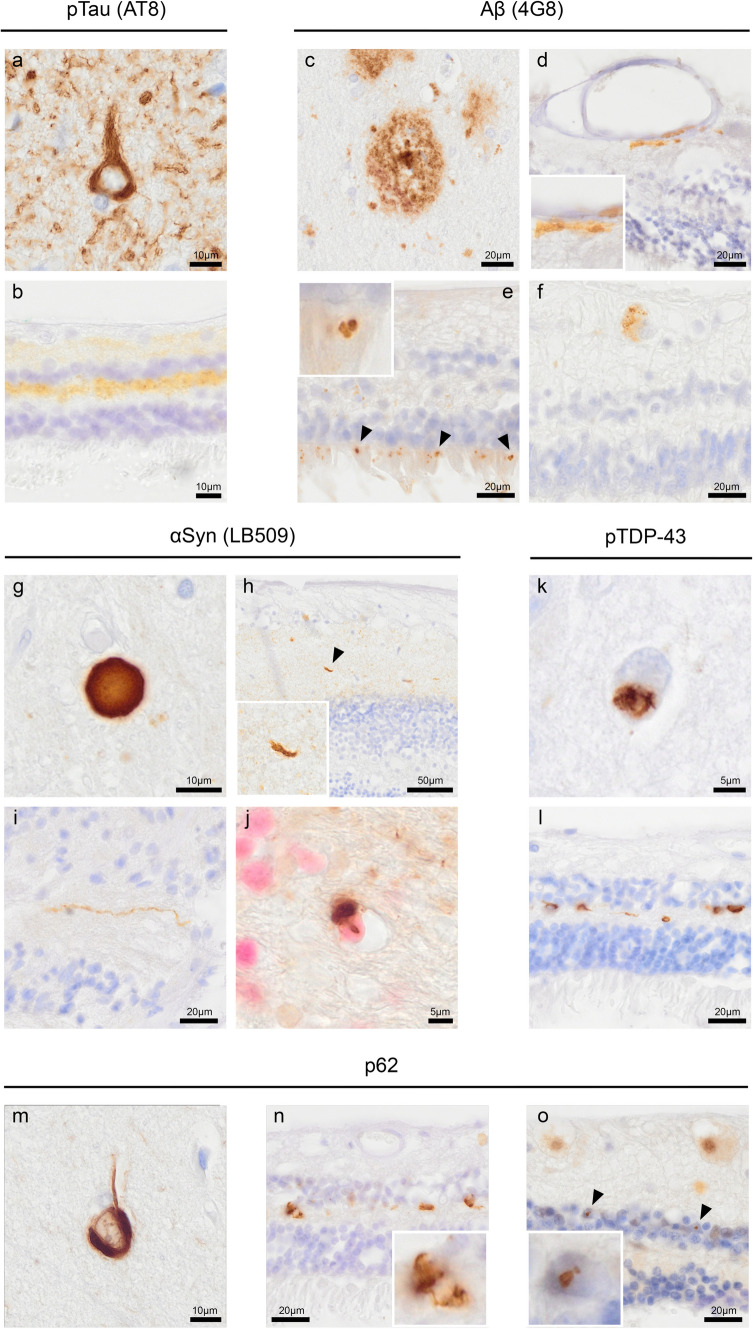
Localization and morphology of pTau, Aβ, αSyn, pTDP-43 and p62 in the retina. The medial frontal gyrus of cases with AD and FTLD-TDP was used as a positive control brain tissue for **a** pTau Ser202/Thr205 (AT8), **c** Aβ (4G8), **k** pTDP-43 and **m** p62. Mesencephalon tissue of a PD case was used as a positive control for **g** αSyn (KM51). **b** pTau was observed as a diffuse signal in the inner and outer plexiform layers of the retina, shown here in an AD case. Aβ was observed as **d** aggregates in the vessel wall in the inner retinal layers, **e** globular deposits in the photoreceptor layer and **f** cytoplasmic granular deposits in the ganglion cells. Anti-αSyn revealed Lewy neurites in **h** the inner plexiform layer of the retina and **i** the optic nerve, with the latter white matter tissue also revealing **j** oligodendroglial cytoplasmic inclusions with anti-SOX-10 labelling oligodendrocyte nuclei (Liquid Permanent Red). **l** pTDP-43 showed typical neuronal cytoplasmic inclusions in the outer plexiform layer. **n** Immunopositive cytoplasmic inclusions were observed with p62 showing overlap with the inclusions observed with anti-pTDP-43. **o** Furthermore, p62 showed intranuclear inclusions in the inner nuclear layer, shown here in a case with neuronal intranuclear inclusion disease. Immunostaining is shown with DAB (brown), and nuclei are counterstained with haematoxylin (blue). In **j**, nuclei are immunolabelled and stained with Liquid Permanent Red

**Fig. 2:**
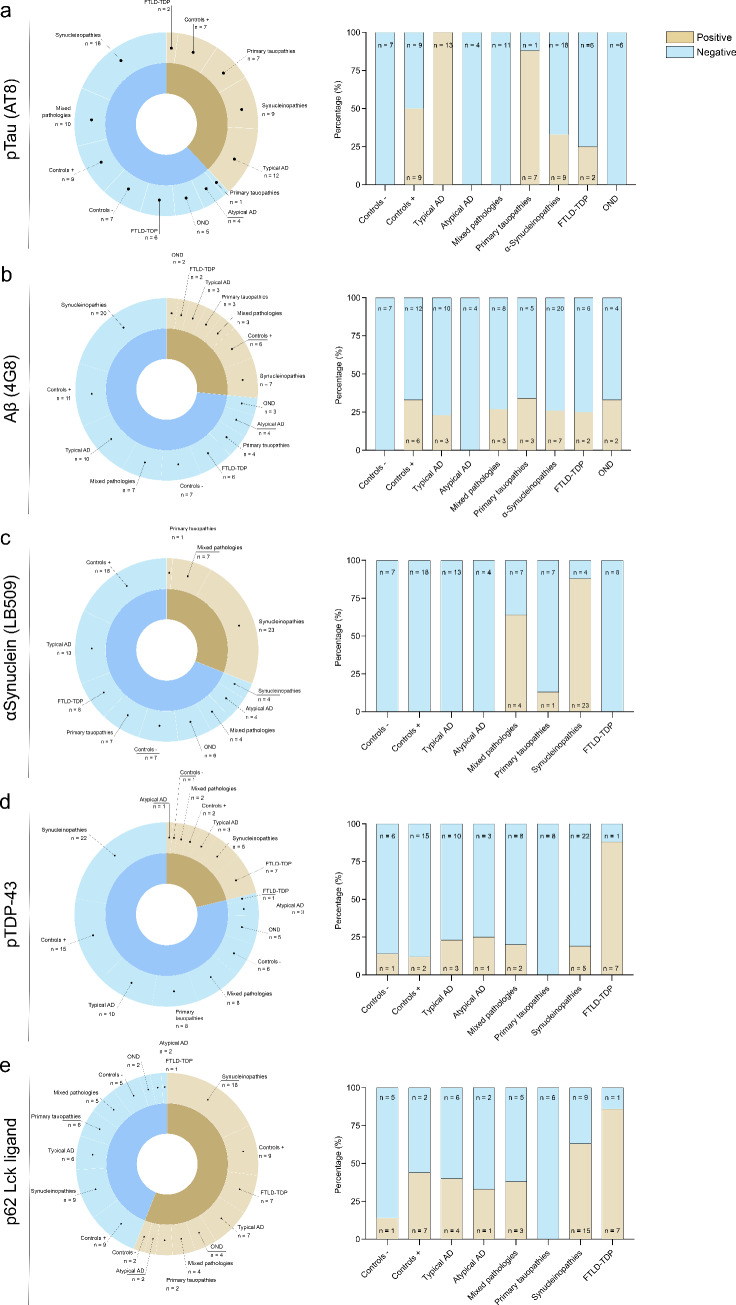
Prevalence of retinal proteinopathies within the different clinicopathological subgroups. The sunburst graphs show the presence (brown) and absence (blue) of proteinopathy and the prevalence in the different subgroups. **a** pTau was present in 39% of the cases and was present in all typical AD cases and most primary tauopathy cases (7/8). Furthermore, pTau was observed in cases with controls with neuropathologic changes (controls +), synucleinopathies and FTLD-TDP-43, all of which showed Braak stages I–III for neurofibrillary tangles. **b** Aβ was present in 26% of all cases and showed variable presence in all groups except controls without neuropathological changes (controls −) and atypical AD. **c** αSyn pathology was present in 30% of all cases and primarily seen in cases with synucleinopathies. Other cases that showed αSyn pathology were synucleinopathies with mixed (tau) pathology (7/11) and one FTLD-TDP-43 case. **d** pTDP-43 pathology was observed in 21% of the cases and was also variably observed in different groups, although the highest incidence was found for FTLD-TDP-43 cases (7/8). **e** p62 generally showed immunopositivity in all groups, in 56% of all cases, and seemed to be specifically associated with FTLD-TPD-43, showing the highest incidence here (7/8). *AD* Alzheimer’s disease; controls −/ + controls without/with neuropathological changes; *FTLD-TDP* frontotemporal lobar degeneration-TAR DNA-binding protein; *OND* other neurodegenerative diseases

**Fig. 3:**
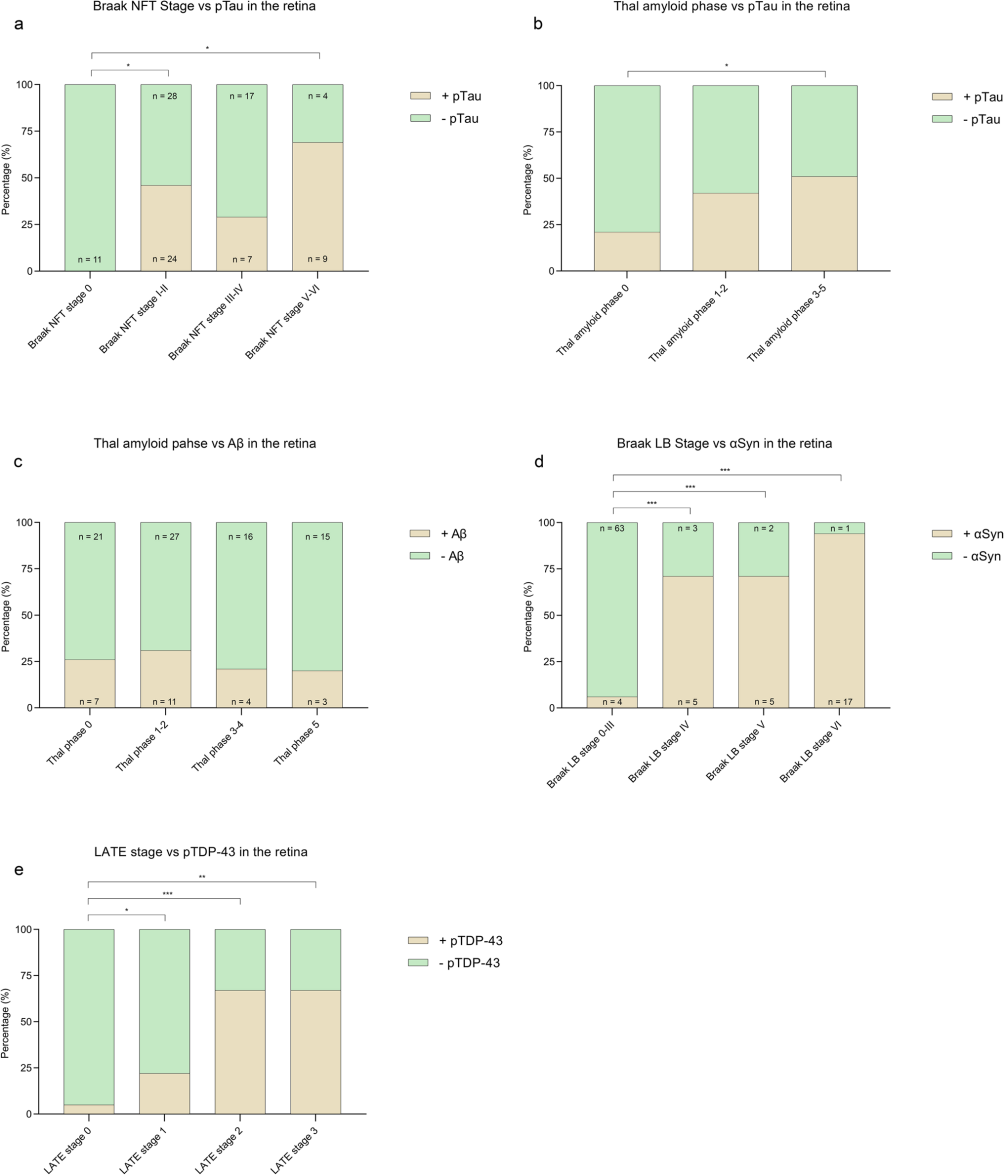
Relation between retinal protein aggregates and neuropathological staging systems. Shown is the percentage of cases with retinal proteinopathy (brown indicates presence, green indicates absence) in the retina nerve associated with neuropathological staging systems. **a** pTau in de retina significantly correlated with Braak NFT stages I–II and V–VI. **b** pTau in the retina showed a positive trend in the association with Thal amyloid phase. **c** No significant association was found between Aβ in the retina and Aβ in the brain. **d** αSyn pathology in the retina significantly correlated with increasing Braak LB stages. **e** pTDP-43 in the retina was significantly associated with LATE stages 2 and 3. *NFT* neurofibrillary tangles; *LB* Lewy bodies; *LATE* limbic predominant age-related TDP-43 encephalopathy

The presence of Aβ, pTau, pTDP-43, αSyn and p62 Lck ligand (p62) was assessed in the retina (Fig. 1). The medial frontal gyrus of cases with Alzheimer’s disease and FTLD-TDP was used as positive control brain tissue for pTau Ser202/Thr205 (Fig. 1a), Aβ (Fig. 1c), pTDP-43 (Fig. 1k) and p62 (Fig. 1m). Mesencephalon tissue of a PD case was used as a positive control for αSyn (Fig. 1g). As reported previously, pTau Ser202/Thr205 showed diffuse immunopositivity (Fig. 1b), primarily in the retina's far- and mid-peripheral region [1, 4]. Immunoreactivity was observed in the outer plexiform layer, where the synapses lie between photoreceptors and retinal cells within the inner nuclear layer, and in the inner plexiform layer, where the synapses lie between retinal cells within the inner nuclear layer and ganglion cells. Structures resembling fibrillary inclusions in the retina were not observed. Aβ immunopositivity was seen (1) as granular structures in the vessel wall in the inner plexiform layer (Fig. 1d), (2) as extracellular, globular structures in all layers, including the photoreceptor layer (Fig. 1e), and (3) as cytoplasmic, granular deposits in the ganglion cell layer (Fig. 1f). Staining for αSyn showed Lewy neurites in the retinal nerve fibre layer, inner plexiform layer (Fig. 1h) and optic nerve head (Fig. 1i). With anti-SOX-10, a nucleus marker for oligodendrocytes (shown in pink), the glial tissue of the optic nerve also showed αSyn immunopositive glial cytoplasmic inclusions (Fig. 1j). Staining for pTDP-43 (Fig. 1l) mainly showed neuronal cytoplasmic inclusions in the inner nuclear layer and short dystrophic neurites in the outer plexiform layer. To a lesser extent, pTDP-43 staining was visible in the inner plexiform layer. Immunopositive inclusions were observed with p62 resembling the neuronal cytoplasmic inclusions seen with anti-pTDP-43 (Fig. 1n). Furthermore, p62 showed small nuclear inclusions in the inner nuclear layer (Fig. 1o).

### Presence of different protein markers across different clinicopathological groups

Protein markers were evaluated across different clinicopathological groups to study the relationship between retina and brain proteinopathy (Fig. 2).

In this study, we report on the absence or presence of retina proteinopathy using a dichotomous scoring system. Additional information on the extent of each proteinopathy is provided in Table 2. pTau positivity was additionally assessed according to the staging for primary retinal tauopathy (PReT staging) [60]. Overall, higher PReT stages (up to stage 3) were observed in typical AD cases and primary tauopathies. Among controls without ADNC, all exhibited a PReT stage of 0, whereas controls with ADNC demonstrated PReT stages ranging from 0 to 3. For tauopathy cases, one case exhibited a PReT stage of 0 (ARTAG). However, for one CBD case, determining the PReT stage was not possible as it displayed a distinctive distribution of pTau, detected with AT8, featuring pTau presence in the IPL but no staining in the OPL. For Aβ and αSyn the number of immunoreactive inclusions observed in the retina mostly remained restricted to a score of few (1–5 positive inclusions). pTDP-43 positive inclusions were scored as many (more than 20 positive inclusions) in most positive cases. As expected, scores for p62 were higher compared to other markers with many cases having either a score for moderate (6–20 inclusions) or many. Apart from the occurrence of pTau, which progresses from the OPL to INL and IPL (following the PReT staging), no relationship was observed between the number of inclusions on the occurrence or spread throughout the retinal layers.

**Table 2:** Dichotomized score of protein presence in brain and retina

Case	Age	Sex	Group	Retinal pathology					Brain pathology			
				pTau (PReT staging)	Aβ (score)	ɑSyn (score)	pTDP-43 (score)	p62 (score)	pTau (AT8)	Aβ (4G8)	ɑSyn (KM51)	pTDP-43
1	33	F	Controls −	− (0)	−	−	−	−	−	−	−	−
2	47	F	Controls −	− (0)	−	−	−	−	−	−	−	−
3	49	M	Controls −	− (0)	−	−	−	−	−	−	−	−
4	55	F	Controls −	− (0)	−	−	−	−	−	−	−	−
5	57	F	Controls −	− (0)	−	−	−	−	−	−	−	−
6	63	F	Controls −	− (0)	−	−	+ (moderate)	+ (many)	−	−	−	−
7	68	M	Controls −	− (0)	−	−	−	+ (many)	−	−	−	−
8	60	F	Controls +	+ (1)	−	−	−	+ (many)	+	+	−	−
9	62	F	Controls +	− (0)	−	−	−	−	+	−	−	−
10	63	F	Controls +	− (0)	−	−	−	+ (few)	+	+	−	−
11	72	F	Controls +	− (0)	+ (few)	−	−	−	+	+	−	−
12	76	F	Controls +	+ (3)	−	−	−	−	+	−	−	−
13	79	F	Controls +	+ (1)	−	−	−	+ (few)	+	−	−	−
14	79	F	Controls +	− (0)	+ (few)	−	+ (few)	+ (few)	+	+	−	+
15	80	M	Controls +	− (0)	+ (few)	−	−	−	+	−	−	−
16	80	M	Controls +	+ (1)	−	−	−	−	+	+	+	−
17	80	F	Controls +	− (0)	−	−	−	−	+	−	−	−
18	81	F	Controls +	− (0)	+ (few)	−	+ (few)	+ (moderate)	+	+	−	−
19	83	F	Controls +	+ (3)	−	−	−	−	+	+	−	−
20	87	M	Controls +	+ (1)	−	−	−	+ (few)	+	−	−	−
21	89	F	Controls +	+ (1)	−	−	−	+ (few)	+	+	−	−
22	89	M	Controls +	+ (3)	+ (few)	−	−	−	+	+	−	−
23	90	M	Controls +	+ (1)	−	−	−	−	+	+	−	−
24	96	F	Controls +	− (0)	−	−	−	−	+	+	+	−
25	98	F	Controls +	− (0)	+ (few)	−	−	+ (few)	+	−	−	−
26	65	M	Typical AD	+ (1)	−	−	−	−	+	+	+	+
27	68	F	Typical AD	+ (1)	−	−	+ (few)	+ (few)	+	+	−	+
28	70	M	Typical AD	+ (1)	−	−	−	−	+	+	−	−
29	71	M	Typical AD	+ (3)	−	−	−	−	+	+	n.a	−
30	73	F	Typical AD	+ (3)	−	−	−	−	+	+	−	−
31	73	M	Typical AD	+ (3)	−	−	−	−	+	+	+	−
32	76	F	Typical AD	+ (1)	+ (few)	−	−	+ (few)	+	+	−	−
33	80	F	Typical AD	+ (1)	−	−	−	−	+	+	−	+
34	82	M	Typical AD	+ (1)	+ (few)	−	−	+ (few)	+	+	+	+
35	82	F	Typical AD	+ (1)	+ (few)	−	+ (few)	+ (moderate)	+	+	+	+
36	89	F	Typical AD	+ (1)	−	−	−	+ (few)	+	+	−	−
37	91	F	Typical AD	+ (3)	−	−	+ (many)	+ (many)	+	+	+	+
38	95	F	Typical AD	+ (1)	−	−	−	+ (few)	+	+	+	−
39	66	F	Atypical AD	− (0)	−	−	+ (many)	+ (many)	+	+	−	−
40	61	F	Atypical AD	− (0)	−	−	−	−	+	+	−	−
41	77	M	Atypical AD	− (0)	−	−	−	−	+	+	−	−
42	72	F	Atypical AD	− (0)	−	−	−	+ (few)	+	+	+	−
43	72	M	Mixed pathology	− (0)	−	+ (few)	−	−	+	+	+	−
44	76	F	Mixed pathology	− (0)	−	−	−	−	+	+	+	−
45	80	M	Mixed pathology	− (0)	−	+ (few)	−	−	+	+	+	+
46	84	M	Mixed pathology	− (0)	−	+ (few)	−	−	+	+	+	+
47	84	M	Mixed pathology	− (0)	−	−	−	−	+	+	+	−
48	86	F	Mixed pathology	− (0)	+ (few)	+ (few)	+ (many)	+ (many)	+	−	+	+
49	86	F	Mixed pathology	− (0)	−	+ (few)	−	−	+	+	+	+
50	91	F	Mixed pathology	− (0)	−	+ (few)	−	−	+	+	+	−
51	92	M	Mixed pathology	− (0)	+ (few)	−	−	−	+	+	+	−
52	93	F	Mixed pathology	− (0)	+ (few)	+ (few)	−	−	+	+	+	−
53	103	F	Mixed pathology	− (0)	−	−	+ (few)	−	+	+	+	+
54	59	M	Primary tauopathies	+ (3)	−	−	−	−	+	+	−	−
55	65	M	Primary tauopathies	− (0)	−	−	−	−	+	+	−	−
56	65	F	Primary tauopathies	+ (3)	+ (few)	−	−	−	+	+	+	−
57	65	M	Primary tauopathies	+ (2)	+ (few)	−	−	−	+	+	−	−
58	71	M	Primary tauopathies	+ (3)	−	+ (moderate)	−	+ (few)	+	+	+	+
59	74	F	Primary tauopathies	+ (n.a.)	−	−	−	−	+	+	−	−
60	80	M	Primary tauopathies	+ (1)	−	−	−	−	+	−	−	−
61	95	F	Primary tauopathies	+ (1)	+ (few)	−	−	+ (moderate)	+	+	−	−
62	57	F	Synucleinopathies	− (0)	+ (few)	+ (few)	−	+ (few)	+	−	+	−
63	57	M	Synucleinopathies	− (0)	−	−	−	+ (few)	−	−	+	−
64	59	F	Synucleinopathies	+ (1)	−	+ (few)	−	+ (few)	+	+	+	−
65	62	F	Synucleinopathies	− (0)	−	−	−	−	+	−	+	−
66	65	M	Synucleinopathies	− (0)	−	+ (few)	−	−	+	−	+	−
67	66	F	Synucleinopathies	+ (1)	−	+ (few)	+ (few)	+ (moderate)	+	−	+	−
68	67	M	Synucleinopathies	− (0)	−	+ (few)	−	+ (few)	+	−	+	+
69	67	F	Synucleinopathies	− (0)	+ (few)	+ (few)	−	−	+	−	+	−
70	72	M	Synucleinopathies	+ (1)	−	+ (few)	−	+ (few)	+	+	+	−
71	72	F	Synucleinopathies	− (0)	−	+ (many)	−	+ (few)	+	+	+	−
72	72	M	Synucleinopathies	+ (1)	−	+ (moderate)	−	+ (few)	+	+	+	−
73	72	M	Synucleinopathies	− (0)	−	+ (few)	−	−	+	+	+	−
74	73	M	Synucleinopathies	− (0)	+ (few)	+ (many)	−	−	+	+	+	−
75	74	M	Synucleinopathies	− (0)	−	+ (few)	−	+ (few)	+	+	+	+
76	75	M	Synucleinopathies	− (0)	+ (few)	+ (few)	−	−	+	+	+	−
77	75	M	Synucleinopathies	− (0)	−	+ (few)	−	+ (few)	+	+	+	−
78	75	M	Synucleinopathies	− (0)	−	+ (few)	+ (few)	+ (few)	+	+	+	+
79	76	M	Synucleinopathies	+ (1)	−	+ (few)	+ (few)	+ (few)	+	+	+	+
80	77	M	Synucleinopathies	− (0)	+ (few)	+ (few)	−	+ (few)	+	+	+	−
81	78	M	Synucleinopathies	+ (1)	+ (few)	−	−	−	+	−	+	−
82	78	M	Synucleinopathies	+ (1)	−	+ (few)	−	−	+	+	+	−
83	79	F	Synucleinopathies	+ (1)	+ (few)	+ (moderate)	+ (few)	+ (moderate)	+	+	+	+
84	80	M	Synucleinopathies	− (0)	−	−	−	+ (few)	+	+	+	−
85	81	F	Synucleinopathies	− (0)	−	+ (few)	−	−	+	+	+	−
86	81	M	Synucleinopathies	− (0)	−	+ (few)	+ (few)	+ (moderate)	+	+	+	+
87	88	F	Synucleinopathies	+ (1)	−	+ (few)	−	+ (few)	+	+	+	+
88	91	F	Synucleinopathies	− (0)	−	+ (few)	−	+ (few)	+	+	+	−
89	51	F	FTLD − TDP	− (0)	−	−	+ (few)	−	−	+	−	+
90	58	M	FTLD − TDP	− (0)	−	−	+ (many)	+ (moderate)	−	−	−	+
91	65	M	FTLD − TDP	− (0)	−	−	+ (many)	+ (many)	+	+	−	+
92	70	M	FTLD − TDP	− (0)	−	−	+ (moderate)	+ (many)	+	+	−	+
93	70	F	FTLD − TDP	+ (1)	+ (few)	−	−	+ (moderate)	+	+	−	+
94	72	M	FTLD − TDP	− (0)	+ (few)	−	+ (few)	+ (few)	n.a	n.a	n.a	+
95	74	F	FTLD − TDP	− (0)	−	−	+ (many)	+ (many)	+	+	−	+
96	75	M	FTLD − TDP	+ (1)	−	−	+ (few)	+ (few)	+	+	−	+
97	50	M	OND	− (0)	+ (few)	−	−	+ (few)	−	−	−	+
98	54	M	OND	− (0)	−	−	−	−	+	−	−	−
99	70	F	OND	− (0)	−	−	−	−	+	−	−	−
100	71	M	OND	− (0)	−	−	−	−	+	+	−	+
101	80	M	OND	− (0)	+ (few)	−	−	+ (few)	+	+	−	−
102	84	M	OND	− (0)	−	−	−	+ (few)	+	+	−	−

Immunoreactivity for pTau was assessed following the staging for primary retinal tauopathy (PReT) [60]. Inclusions immunoreactive for αSyn, 4G8, pTDP-43 and p62 were scored as follows: few for 1–5 inclusions, moderate for 6–20 inclusions, many for more than 20 inclusions along the entire cross-section of the retina (from far peripheral to central retina)

*AD* Alzheimer’s disease; *FTLD* frontotemporal lobar degeneration; *OND* other neurodegenerative diseases; *n.a*. not available. *Controls − *, without neuropathological changes; *controls* + , with neuropathologic changes

In total, 42% of all cases showed retinal pTau positivity (Fig. 2a). A pTau immunopositive signal in the retina was seen in all 13 typical AD cases (OR = 375.0, *P* = 0.004) and a large proportion of the primary tauopathies (OR = 75.0, *P* = 0.01). In cases that showed incidental tau pathology in the brain, such as some of the controls, synucleinopathies and FTLD-TDP cases, pTau was also found in the retina, as opposed to none of the controls without incidental pathology. Furthermore, in none of the four atypical AD cases pTau was observed. Similarly, in the 11 cases with mixed pathology, where advanced AD coincided with Lewy body pathology and primary supranuclear palsy pathology coincided with Lewy body pathology, no retinal pTau was observed (Supplemental material 1, Table 2).

Aβ-positive structures were observed in 20% of all cases and in all groups, except controls without pathology and atypical AD (Fig. 2b). No significant associations were observed between retinal Aβ and clinicopathological groups. Three out of 13 typical AD cases showed retinal Aβ. Notably, retinal Aβ depositions were found in seven cases without Aβ in the brain (Thal phase 0) (Fig. 3c). Finally, assessing the relation between Aβ in the retina and cerebral amyloid angiopathy revealed no significant associations.

In 30% of all cases and in 88% of all synucleinopathies (OR = 100.71, *P* = 0.003), there was evidence of αSyn pathology in the retina/optic nerve (Fig. 2c). This resulted in a sensitivity of 83% and a specificity of 98% for predicting the presence of synucleinopathy in the brain. Additionally, retinal αSyn pathology was also observed in cases with mixed pathology (OR = 25.0, *P* = 0.041) and in one FTLD-tau case (#58), all having moderate to advanced stages of synucleinopathy (Braak LB stage ≥ 3). Not all cases with αSyn pathology in the brain also showed αSyn pathology in the retina. Four out of 27 (15%) synucleinopathy cases (#63, #65, #81, #84) and two out of nine (30%) mixed pathology cases with advanced synucleinopathy mixed with AD (#44) or progressive supranuclear palsy cases (#51) did not show retinal αSyn pathology. Additionally, 13 cases showed limbic or amygdala-only Lewy pathology, yet none of them showed retinal αSyn pathology. Also, all seven cases with clinical multiple system atrophy only showed glial cytoplasmic inclusions in the optic nerve and no Lewy neurites in the retina, despite Lewy copathology in the brain. Finally, none of the 50 cases without Lewy pathology in the brain showed Lewy pathology in the retina or optic nerve.

pTDP-43 inclusions were observed in 21% of all cases, mainly in FTLD-TDP cases (OR = 21.67, *P* = 0.020) (Fig. 2d). One of eight FTLD-TDP cases did not show pTDP-43 in the retina (#93). Notably, in brain pTDP-43 negative cases, retinal pTDP-43 pathology was generally absent (89%). However, retinal pTDP-43 was observed in three cases (#18, #39, #67) and one control without pathology (#6), without pTDP-43 observed in the hippocampus or amygdala.

Finally, in this cohort, one of the donors (#102) had nuclear intracellular inclusion disease associated with Fragile X syndrome and showed p62-positive nuclear inclusions in the inner nuclear layer (Fig. 1o). In most groups, p62-positive structures were detected, particularly in cases with FTLD-TDP (Fig. 2e). Five of these cases showed cytoplasmic inclusions, and one showed structures that resembled corpora amylacea.

### Retinal proteinopathy is associated with corresponding brain pathology

The presence of retinal proteinopathy was correlated to pathological staging systems (Fig. 3). A significant association was found between retinal pTau and Braak NFT stage I–II (OR = 20.49, *P* = 0.040) and V–VI (OR = 48.56, *P* = 0.012) (Fig. 3a). To study whether retinal pTau was present as part of ADNC, the relationship between retinal pTau with Thal amyloid phase was assessed, yielding a positive trend with increasing Thal amyloid phase (Thal amyloid 1–2: OR = 2.66, *P* = 0.08; Thal amyloid phase 3–5: OR = 3.66, *P* = 0.02) (Fig. 3b). No significant association was found between Aβ and Thal amyloid phase (Fig. 3c). Furthermore, the likelihood of finding αSyn in the retina significantly increased with Braak LB stage, with the highest odds ratio found for Braak LB stage VI (OR = 143.89, *P* < 0.001) (Fig. 3d). LATE-NC with varying stages from 1 to 3 was found in 21 cases, of which 10 showed pTDP-43 in the retina (Fig. 3e). The likelihood of finding pTDP-43 in the retina was significantly increased with LATE-NC stages 2 (OR = 14.59, *P* = 0.002) and 3 (OR = 13.10, *P* < 0.03). The presence of pTau immunoreactivity and the occurrence of inclusions immunopositive for Aβ, pTDP-43, αSyn, and p62 showed a prevalence for the peripheral retina. The predominant occurrence between the peripheral and central regions did not change with increased occurrence of immunoreactivity or immunopositive inclusions or corresponding brain pathology.

### The retina does not mirror the heterogeneity of protein aggregation in the brain

**Fig. 4:**
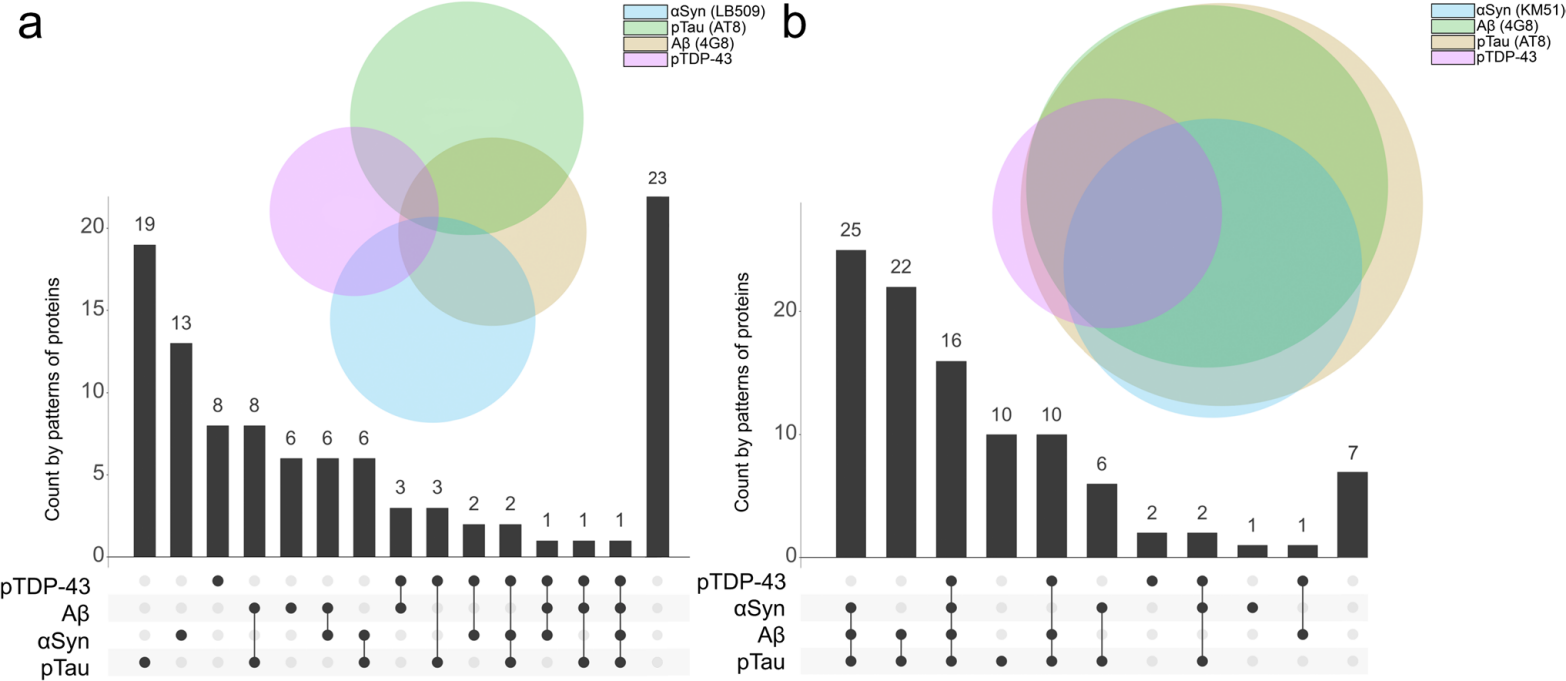
Proteinopathy heterogeneity in the brain is larger than in the retina. The upset plots show the number of cases on the *y*-axis that have a specific combination of proteins depicted on the *x*-axis. The Venn diagrams represent the number of cases with one specific protein (in colours). The overlapping circles in the Venn diagrams show less co-occurrence of proteins in the retina (**a**) than in the brain (**b**). In the retina, pTau (*n* = 19) and αSyn (*n* = 13) are most often observed solely, whereas, in the brain, most cases show copathology of pTau, Aβ and αSyn without pTDP-43 (*n* = 25). In the retina, only one case showed the presence of all four proteins. The sizes of the circles reflect the number of cases in which a certain protein is observed. In the retina, pTau (green) and αSyn pathology (blue) are most often observed. In brain tissue, the most common pathological proteins observed are pTau (brown) and Aβ (green). pTDP-43 (pink) was shown to be the least prevalent in the retina and brain

**Fig. 5:**
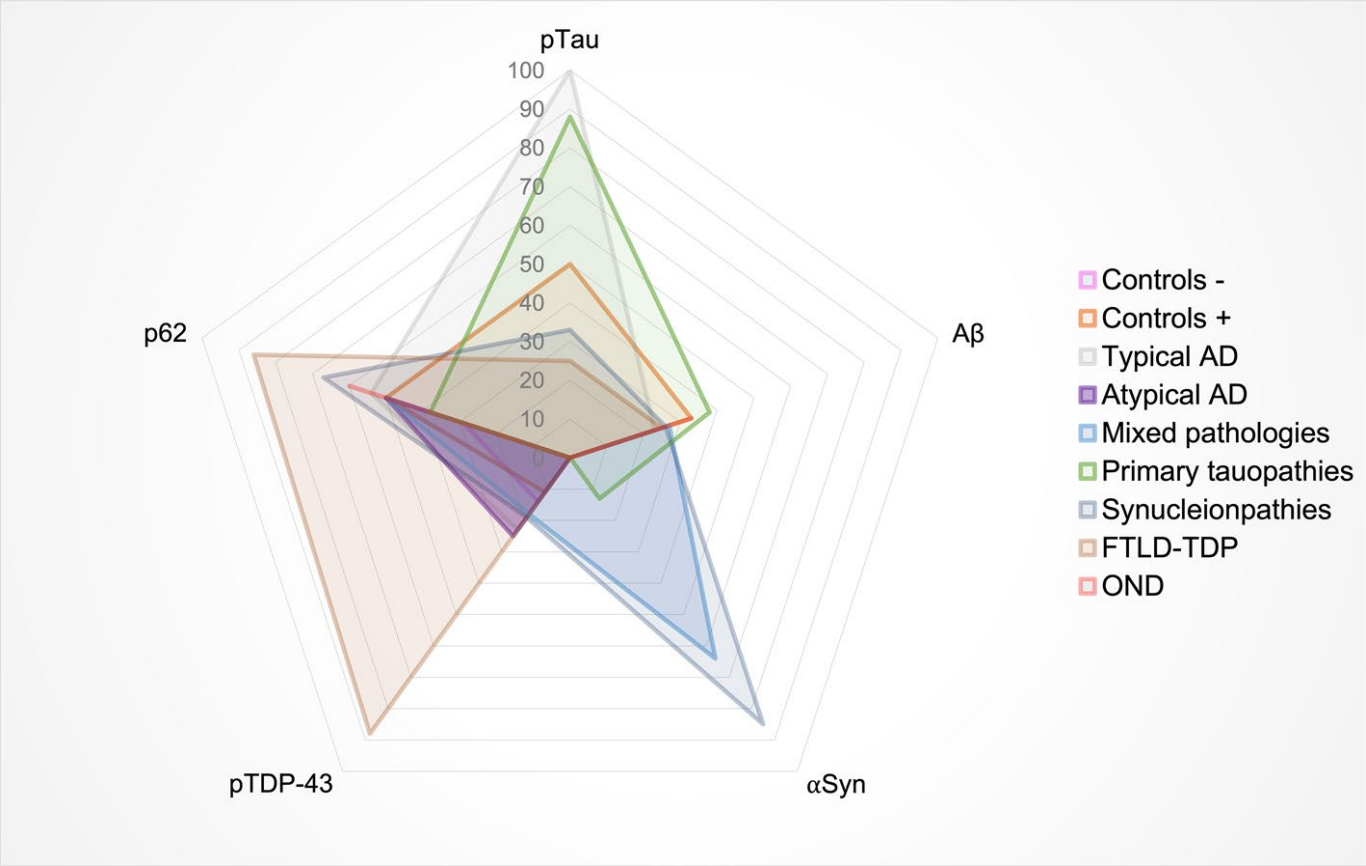
Proteinopathy distribution across different cliniconeuropathological groups. The spider plot illustrates the spread of the different pathological proteins within the retina across the major clinicopathological groups. Data are shown in percentages of cases in which the proteins were observed. Each step towards the outside of the plot represents 10% with a maximum of 100%. The legend shows the cliniconeuropathological groups depicted in different colours. Controls with pathology (orange) showed co-presence of p62 (50%), Aβ (35%), pTau (50%) and pTDP-43 (11%). In typical AD (light grey), retinal pTau is observed in 100% of the cases and co-occurs with p62 in 55% and with Aβ in 25% of these cases. Atypical AD cases (purple) showed p62 positivity (50%), together with some pTDP-43 inclusions (25%). The mixed pathology group cases (blue) showed retinal αSyn pathology in 64%, p62 positivity in 45%, Aβ in 27% and pTDP-43 in 18%. Primary tauopathies (green) showed pTau in nearly 90%, occurring mainly with Aβ (38%) and p62 (30%). Synucleinopathies (dark grey) showed mostly αSyn pathology in the retina (90%), and also showed a prevalence of p62 (55%), pTau (33%), Aβ (26%) and pTDP-43 (19%). FTLD-TDP (brown) showed evenly co-occurrence of pTDP-43 and p62 with 88% and in some cases co-occurrence with pTau and Aβ (25%)

To further investigate if the heterogeneity of protein pathology in the brain was mirrored by the retina, the presence of pTau, Aβ, αSyn and pTDP-43 was compared between the two tissues (Fig. 4). The prevalence of protein deposits in the brain was more heterogeneous than in the retina. pTau and αSyn were most frequently observed in the retina without other coinciding pathology. pTau and Aβ were more prevalent in the brain than the other two proteins. pTDP-43 was the least prevalent in both the retina and brain. Where pathological proteins were most often observed solely in the retina, in the brain, most cases showed copathology of pTau, Aβ with αSyn (*n* = 25) or without αSyn (*n* = 22). Only one case showed all four proteins in the retina (#83). The spider plot in Fig. 5 illustrates the distribution of the different protein aggregates within the retina across the different major clinicopathological groups. In typical AD cases, pTau was present in all cases. This group also showed p62-positivity, and few cases also exhibited Aβ pathology. In atypical AD cases, p62 positivity (50%) and, in one case, pTDP-43 inclusions (25%) were observed. Primary tauopathies mainly showed pTau (90%). αSynucleinopathies showed mostly αSyn pathology in the retina (90%), and additionally p62 positivity (65%). In contrast, despite all having advanced αSyn pathology, mixed pathology cases were less frequently positive for αSyn pathology in the retina. FTLD-TDP showed equal co-occurrence of pTDP-43 and p62 positivity (85%). Controls with pathology showed co-presence of p62 (50%), Aβ (35%), pTau (50%) and pTDP-43 (10%).

## Discussion

This study aimed to understand how protein accumulation in the retina is related to neurodegenerative proteinopathy in the brain and how the retina mirrors the primary neurodegenerative disease of the brain. Insight in the co-occurrence of proteinopathy in the retina is crucial for development of retinal biomarkers [5]. Findings from this study suggest that the retina is a site in the central nervous system that reflects the primary proteinopathy in major neurodegenerative diseases. Moderate co-occurrence of pathological proteins observed in the brain is not mirrored within the retina. These results from a cross-disease post-mortem cohort strengthen the retina's possible role as a specific and sensitive source for biomarkers predicting typical AD, primary tauopathies, synucleinopathies, and cortical TDP-43 (as seen in FTLD-TDP).

This study indicates that the four hallmark proteins underlying the major neurodegenerative diseases are also found within the retina. However, morphological differences exist between the two neuronal tissues. In this study, pTau was observed as a diffuse signal without revealing neurofibrillary tangles [17, 21], and αSyn aggregates were observed as Lewy neurites without structures resembling Lewy bodies. The chaperone protein p62 was used as a marker for intracellular protein deposition and is abundantly present in diverse pathological inclusions compromising both cytoplasmic and nuclear inclusions with abnormal tau, TDP-43, or αSyn [33]. The lack of immunopositivity for p62 in sequential cross-sections resembling tangles or Lewy bodies supported their absence in the retina. Similarly, the sporadic αSyn-positive ganglion cells and the structures in the optic nerve resembling glial cytoplasmic inclusions in the brain could not be detected with p62. The first may previously have been described as Lewy bodies in whole-mount sections of the retina [20]. Although Lewy bodies were not detected, Lewy neurites were clearly distinguishable in the retina and the optic nerve. Furthermore, pTDP-43 inclusions in the retina were similar to those seen in brain tissue, and the presence of cellular inclusions was confirmed using p62. In addition, nuclear inclusions such as those observed in the brain of nuclear intracellular inclusion disease were observed within the nuclei of the inner nuclear layer, and multiple system atrophy cases showed glial cytoplasmic inclusions in the optic nerve similar to those observed in the brain white matter. The morphological observations for retinal pTau, αSyn and pTDP-43 are in concordance with previous studies [11, 17, 20, 21, 46]. In contrast, the presence of Aβ in the post-mortem retina has been a subject of controversy due to conflicting results among various studies. In support of the harmonization of protocols, we utilized the 4G8 antibody to detect Aβ in the retina, following the guidelines provided by Brain Net Europe [1–4]. In this study, Aβ was mainly observed as small dot-like deposits in the vessel wall and as granular, cytoplasmic immunopositivity in the ganglion cell layer. No structures resembling typical extracellular plaques, as seen in the brain, were observed. Although Aβ-positive structures were not observed in control cases without neuropathologic changes, they were observed in all other groups and were not primarily associated with Alzheimer's disease. These findings concord with earlier findings using the 4G8 antibody [17, 54]. Recently, retinal Aβ using multiple immunohistochemical stainings (4G8, Congo Red and Thioflavin S stains) was studied in a large cohort, including AD and control cases [60]. In only three out of 17 AD cases, small Aβ spots were observed and no fibrillar amyloid was detected. It is hypothesised that the cytoplasmic staining in the ganglion cell reflects the expression of amyloid precursor protein as part of metabolic activity in retinal homeostasis [17, 56]. Our observation of retinal Aβ immunopositivity in cases without amyloid in the brain (Thal amyloid 0) supports this hypothesis and further argues against AD-specific Aβ accumulation in the retina. The absence of Aβ-plaques and lack of specificity for AD in this study contradict earlier findings in whole-mount retinas of patients with AD. [12, 13, 29, 30, 38, 55] These differences between studies may be attributed to tissue preparation (fixation, cross-sections versus whole-mounts) or the use of different Aβ antibodies.

Interestingly, AD cases with an atypical progression of NFT pathology were found negative for pTau in the retina [40]. pTau was predominantly present in typical AD and primary tauopathies. This suggests that the retina may participate in the hierarchical propagation of pTau in the brain, and its involvement may vary across different neuropathological phenotypes of AD. The significant association between retinal pTau with Braak NFT stage V-VI suggests that the retina is more implicated when brain proteinopathy is present in cortical regions. However, pTau was also observed in control cases with only subcortical pTau [32]. Therefore, it remains unclear if pTau in the retina predicts the progression to neurodegenerative pathology and if it can discriminate disease neuropathological changes from ageing-related changes that do not affect the clinical phenotype. Interestingly, the molecular composition of retinal tau has been demonstrated to differ from that of other tauopathies. In the retina, pTau primarily manifests as tau species of 55 kDa or less, and notably lacks an argyrophilic nature, as evidenced by negative Gallyas staining in pTau immunoreactive retinas. These distinctive characteristics suggest that the presence of pTau may be indicative of a primary retinal tauopathy [60].

In this study, we observed a strong relationship between retinal αSyn pathology and the presence of a primary synucleinopathy in the brain. Previous immunohistochemical analyses have already demonstrated the presence of αSyn aggregates in the retinas of individuals with synucleinopathies [6, 20, 46, 59]. Interestingly, a positive correlation between retinal αSyn levels with brain synucleinopathy levels and disease severity was observed [46], which further supports a direct link between pathology in the brain and corresponding proteinopathy in the retina. Additionally, retinal αSyn remains highly sensitive and specific in this multi-cross-pathology study, making αSyn pathology in the retina a potential biomarker for diagnosing synucleinopathies in a clinical setting. In our previous report, we noted an increased occurrence of visual hallucinations in cases with αSyn pathology affecting the retina/optic nerve [20]. Given the complex nature of these hallucinations, it is probable that their cause lies in cortico-cerebral αSyn pathology rather than ophthalmologic αSyn pathology. In this study, we did not find any association between visual impairments and the presence of other pathological inclusions we examined. Detecting such an association would necessitate larger sample sizes and well-documented data on visual impairments. Furthermore, to comprehend the link between complex visual impairments and retinal pathology, future studies should investigate the possibility of hierarchical spreading of pathology along the retino-geniculo-cortical pathway.

Immunostaining with pTDP-43 showed inclusions in seven out of eight cases with FTLD-TDP. pTDP-43 in the retina was strongly associated with FTLD-TDP as well as advanced stages of LATE-NC. pTDP-43 was absent in the retina in LATE-NC stages 0–1. The apparent association between retinal pTDP-43, FTLD-TDP and LATE-NC stage 2 (hippocampus) to 3 (medial-temporal cortex) as opposed to amygdala-associated pTDP-43 suggests that proteinopathy in the retina is mainly involved when widespread, cortical pathology is present. Caution is warranted when interpreting the results concerning the association with LATE-NC stage 3, given the very small number of cases in this study. Future studies should address this relationship in larger cohorts. Retinal pTDP-43 was also found in three cases without (sub)cortical pTDP-43. According to clinical history records, one of these cases had severe head trauma (#39), one had macular Pucker (#18), and one had an extensive ophthalmologic disease history (#67) (Supplemental Table 1). Possibly, pathological pTDP-43 protein deposition could be induced by local inflammation or traumatic impact. Recently, it was shown that retinal pTDP-43 pathology can be observed in cases with chronic traumatic brain injury [49].

The co-occurrence of proteinopathy in the brain increases with age. For diagnostic and therapeutic purposes, it is crucial to discriminate between ageing and neurodegenerative-related proteinopathy [15, 32, 51]. No association was observed between retinal pTau and minimal presence of ARTAG or PART [31]. Regarding αSyn, the presence of limbic synucleinopathy did not seem to impact the involvement of the retina. According to the current literature, early-stage pathological changes in the brain are considered incidental pathologies, and it remains unclear what their clinical relevance is and whether they will progress into severe neurodegenerative pathology [15]. It is important to remark that the cases in this study presented one main neurodegenerative disease sometimes accompanied by low to moderate copathology. Future studies should address the presence of copathology in the retina of cases with advanced presence of different primary pathologies in the brain (e.g., neurofibrillary tangles and Lewy bodies), as this could contribute to our understanding of the involvement of the retina in these diseases.

Certain limitations need to be considered. Cross-sections could result in sampling bias, potentially leading to missing pathology. In addition, larger surface areas of brain tissue assessed, compared to the significantly smaller area of retinal tissue, could cause discrepancies in the findings. Another limitation is that, for the detection of different proteinopathies, we utilised a single antibody to identify immunoreactivity or inclusions associated with each proteinopathy. It should be acknowledged that using various antibodies targeting pTau, may offer different degrees of sensitivity or selectivity in detecting tau pathology. Previous research has shown that pTau Thr217 demonstrates high selectivity for immunostaining retinal pTau in tauopathies. Conversely, antibodies targeting pTau Thr181 and Ser396 may provide high sensitivity in detecting pTau in the retina, although they may not distinctly differentiate between control cases and disease groups [21].

So far, this study contains the largest combined post-mortem cohort of retinas and brain tissue of the major neurodegenerative diseases. Herewith, we aimed to gain a deeper understanding of retinal proteinopathy associated with neurodegenerative brain diseases, which can ultimately help develop a diagnostic tool. The assessment of multiple proteins in this study and the interrelation between neurodegenerative pathologies gives insight into the retina's involvement in these pathologies. Findings from this study show that retinal proteinopathy is more closely associated with the primary neurodegenerative diseases than incidental and ageing-related pathology. With the growing evidence and acknowledgement of the potential of retinal biomarkers and the development of retinal imaging techniques, it is important to consider the specificity and selectivity of proteinopathy in the retina. Although in vivo research and validation are needed to establish the diagnostic value of retinal proteinopathy, results from this study hold promise for the development of non-invasive and accessible methods for the (early) detection and monitoring of AD, synucleinopathies and TDP-43-related disorders.

### Supplementary Information

Below is the link to the electronic supplementary material.

Supplementary file1 (PDF 397 KB)
