# Supplementary material for: Neuropathological hallmarks in the post-mortem retina of neurodegenerative diseases
Source: Acta Neuropathol. 2024 Aug 19;148(1):24. doi: 10.1007/s00401-024-02769-z (PMC11333524; doi:10.1007/s00401-024-02769-z)
Supplement: Supplementary file 1 — Supplementary file1 (PDF 397 KB) [file 401_2024_2769_MOESM1_ESM.pdf]

Supplemental tables

Supplemental Table 1: Cohort characteristics of cases in this study.

Case	Age	Sex	Ocular history	Primary clinicopathological diagnosis	Group	Braak NFT stage	Thal phase	Braak LB stage	LATE stage (0-3)	CAA (type/stage)	ARTAG	PART	CERAD
1	33	F	-	Control	Controls -	0	0	0	0	-	-	-	0
2	47	F	-	Control	Controls -	0	0	0	0	-	-	-	0
3	49	M	-	Control	Controls -	0	0	0	0	-	n.a.	n.a.	0
4	55	F	-	Control	Controls -	0	0	0	0	-	-	-	0
5	57	F	-	Control	Controls -	0	0	0	0	1/2	n.a.	n.a.	0
6	63	F	-	Control	Controls -	0	0	0	0	-	n.a.	n.a.	0
7	68	M	-	Control	Controls -	0	0	0	0	-	-	n.a.	0
8	60	F	-	Control	Controls +	1	1	0	0	2/1	-	-	0
9	62	F	Retinal detachement (2007, 2009)	Control	Controls +	1	0	0	0	-	-	n.a.	0
10	63	F	Glaucoma acute (1990/2015)	Control	Controls +	2	1	0	0	2/1	-	-	0
11	72	F	-	Control	Controls +	3	1	0	0	2/1	-	yes	0
12	76	F	-	Control	Controls +	2	0	0	0	-	yes	yes	0
13	79	F	-	Control	Controls +	1	0	0	0	-	n.a.	n.a.	0
14	79	F	-	Control	Controls +	3	3	0	2	2/1	++	-	2
15	80	M	-	Control	Controls +	2	0	0	0	-	n.a.	yes	0
16	80	M	Macular degeneration	Control	Controls +	2	4	0	0	-	n.a.	n.a.	0
17	80	F	-	Control	Controls +	3	0	0	0	-	yes	yes	0

18	81	F	Macular pucker	Control	Controls +	2	1	0	0	-	-	-	0
19	83	F	-	Control	Controls +	1	3	0	0	1/3	-	n.a.	0
20	87	M	-	Control	Controls +	2	0	0	0	-	n.a.	n.a.	0
21	89	F	-	Control	Controls +	2	2	0	0	2/1	n.a.	n.a.	0
22	89	M	-	Control	Controls +	1	1	0	0	2/2	-	-	0
23	90	M	-	Control	Controls +	2	1	0	0	-	-	-	1
24	96	F	-	Control	Controls +	2	1	4	0	2/1	-	-	0
25	98	F	Macular degeneration (dry) (2010), cataract (2011)	Control	Controls +	2	0	0	0	-	+	+	0
26	65	M	-	AD	Typical AD	5	4	0	1	1/2	+	n.a.	3
27	68	F	-	AD	Typical AD	5	4	0	3	2/1	yes	-	2
28	70	M	-	AD	Typical AD	6	5	0	0	-	n.a.	n.a.	0
29	71	M	-	AD	Typical AD	4	4	n.a.	0	-	n.a.	n.a.	1
30	73	F	Retinal detachement	AD	Typical AD	6	5	0	0	-	n.a.	n.a.	3
31	73	M	-	AD	Typical AD	6	5	0	0	1/1	+	n.a.	3
32	76	F	-	AD	Typical AD	5	5	0	0	1/2	-	-	3
33	80	F	-	AD	Typical AD	4	5	0	1	2/1	+++	n.a.	2
34	82	M	-	AD	Typical AD	5	5	0	3	2/1	-	n.a.	3
35	82	F	-	AD	Typical AD	5	5	0	1	1/1	++	n.a.	3
36	89	F	-	AD	Typical AD	4	3	0	0	-	n.a.	n.a.	1
37	91	F	-	AD	Typical AD	5	4	0	2	1/2	n.a.	n.a.	3

38	95	F	-	AD	Typical AD	4	3	3	0	2/1	yes	-	2
39	66	F	-	AD	Atypical AD	6	5	0	0	2/1	+/++	n.a.	3
40	61	F	-	AD	Atypical AD	6	5	0	0	1/1	-	n.a.	3
41	77	M	-	AD	Atypical AD	4	5	0	0	2/1	+	n.a.	2
42	72	F	-	AD	Atypical AD	6	5	0	0	2/1	-	-	3
43	72	M	-	DLB	Mixed pathology	3	4	6	0	1/3	-	-	2
44	76	F	-	MSA-c/p	Mixed pathology	4	3	5	0	-	n.a.	n.a.	0
45	80	M	-	AD	Mixed pathology	6	5	4	1	1/2	++	n.a.	3
46	84	M	-	MSA	Mixed pathology	4	5	4	1	1/2	+++	-	1
47	84	M	-	AD	Mixed pathology	4	5	3	0	-	+	n.a.	2
48	86	F	-	PDD	Mixed pathology	1	0	5	3	-	+++	n.a.	0
49	86	F	-	DLB	Mixed pathology	4	5	6	2	1/2	-	-	2
50	91	F	-	DLB	Mixed pathology	3	3	6	0	2/1	n.a.	n.a.	1
51	92	M	Macular degeneration (dry) (2017), cataract (2012), vitrectomy OD (2012)	PD	Mixed pathology	4	3	5	0	2/1	+++	-	1
52	93	F	-	PDD	Mixed pathology	3	3	6	0	1/1	+++ (AP)	-	1
53	103	F	-	ARTAG	Mixed pathology	3	3	0	2	1/1	+++	n.a.	1
54	59	M	-	CBD	Primary tauopathies	1	2	0	0	2/1	+	-	0
55	65	M	-	FTLD Tau	Primary tauopathies	n.a.	1	0	0	2/1	-	n.a.	0
56	65	F	-	PSP	Primary tauopathies	2	3	0	0	-	n.a.	n.a.	1

57	65	M	-	PSP	Primary tauopathies	2	1	0	0	-	-	n.a.	0
58	71	M	-	FTLD Tau (Pick's disease)	Primary tauopathies	2	2	3	1	-	-	-	0
59	74	F	-	CBD	Primary tauopathies	1	3	0	0	-	n.a.	n.a.	0
60	80	M	-	PART/ARTAG	Primary tauopathies	2	0	0	0	-	+	+	0
61	95	F	-	PART/ARTAG	Primary tauopathies	3	1	0	0	2/1	+	++	0
62	57	F	-	MSA-p	Synucleinopathies	1	0	4	0	-	-	-	0
63	57	M	-	PD	Synucleinopathies	0	0	4	0	-	-	-	0
64	59	F	-	MSA-p	Synucleinopathies	2	1	3	0	-	-	-	0
65	62	F	-	PD	Synucleinopathies	1	0	4	0	-	n.a.	n.a.	0
66	65	M	-	PD	Synucleinopathies	1	0	3	0	-	-	-	0
67	66	F	Glaucoma (1996), recurrent eye inflammation (syndrome of Cogan), recurrent scleritis (2017), recurrent optic nerve inflammation (2018), cataract (2012)	PD	Synucleinopathies	1	0	6	0	-	-	-	0
68	67	M	-	PDD	Synucleinopathies	3	0	6	2	-	-	++	0
69	67	F	-	MSA-c/p	Synucleinopathies	2	0	5	0	-	yes	-	0
70	72	M	-	PD	Synucleinopathies	2	1	6	0	-	-	n.a.	0
71	72	F	Glaucoma	MSA-p	Synucleinopathies	2	2	3	0	-	+	-	0
72	72	M	-	MSA-p	Synucleinopathies	1	1	5	0	1/1	-	-	0

73	72	M	-	PD	Synucleinopathies	1	1	6	0	-	-	n.a.	0
74	73	M	-	PDD	Synucleinopathies	3	1	6	0	2	yes	-	0
75	74	M	-	PDD	Synucleinopathies	3	2	6	2	2/1	-	+++	0
76	75	M	-	PD	Synucleinopathies	1	2	6	0	1/1	-	n.a.	0
77	75	M	-	PDD	Synucleinopathies	2	3	6	0	1/1	-	-	0
78	75	M	-	DLB	Synucleinopathies	1	3	6	2	2/1	-	-	0
79	76	M	-	PDD	Synucleinopathies	3	2	6	2	-	n.a.	n.a.	1
80	77	M	-	PD	Synucleinopathies	1	1	6	0	-	-	++	0
81	78	M	Glaucoma	PDD	Synucleinopathies	2	0	6	0	-	+	+	0
82	78	M	-	PDD	Synucleinopathies	2	1	6	0	-	yes	-	0
83	79	F	Glaucoma	PD	Synucleinopathies	2	1	5	2	-	-	-	0
84	80	M	-	PD	Synucleinopathies	2	1	3	0	2/1	-	-	0
85	81	F	-	DLB	Synucleinopathies	1	1	4	0	-	yes	n.a.	0
86	81	M	-	PDD	Synucleinopathies	3	1	6	1	1/1	n.a.	+	0
87	88	F	-	PDD	Synucleinopathies	3	1	5	1	-	++	+	0
88	91	F	-	PDD	Synucleinopathies	1	1	4	0	2/1	n.a.	n.a.	0
89	51	F	-	FTLD-TDP-E (sporadic)	FTLD-TDP	0	1	0	FTLD-TDP	-	-	-	0
90	58	M	-	FTLD-TDP-A (Progranulin)	FTLD-TDP	0	0	0	FTLD-TDP	-	+	n.a.	0
91	65	M	-	FTLD-TDP-B (c9orf)	FTLD-TDP	2	1	0	FTLD-TDP	1/1	-	n.a.	0
92	70	M	-	FTLD-TDP-B (c9orf)	FTLD-TDP	1	1	0	FTLD-TDP	2/1	+	-	0
93	70	F	-	FTLD-TDP-A (c9orf?)	FTLD-TDP	2	1	0	FTLD-TDP	-	-	-	0

94	72	M	Glaucoma	FTLD-TDP-C	FTLD-TDP	n.a.	n.a.	n.a.	FTLD-TDP	-	-	n.a.	-
95	74	F	-	FTLD-TDP-B (c9orf)	FTLD-TDP	2	1	0	FTLD-TDP	-	-	-	0
96	75	M	-	FTLD-TDP-B (c9orf)	FTLD-TDP	2	3	0	FTLD-TDP	-	yes	n.a.	0
97	50	M	-	FTLD FUS	OND	0	0	0	1	-	n.a.	n.a.	0
98	54	M	-	MS	OND	1	0	0	0	-	-	n.a.	0
99	70	F	-	Hydrocephalus adultorum	OND	2	0	0	0	-	-	yes	0
100	71	M	-	ALS	OND	1	1	0	0	-	-	n.a.	0
101	80	M	-	Vascular dementia	OND	2	2	0	0	+	+	-	0
102	84	M	-	FXTAS/FXAND/NIID	OND	2	1	0	0	1/1	+	-	0

Abbreviations: ARTAG, age-related tau astrogliopathy, CBD, corticobasal degeneration, ALS, amyotrophic lateral sclerosis; FTLD, frontotemporal lobar degeneration; DLB, dementia with Lewy bodies; MS, multiple sclerosis; MSA, multiple system atrophy; n.a., not available; NFT, neurofibrillary tangles; PCA, posterior cortical atrophy; PD(D), Parkinson's disease (with dementia); PSP, progressive supranuclear palsy; PART, primary age-related tauopathy; -, absent.

Supplemental Table 2: Primary antibodies used in this study.

Name	Epitope	Source	RRID	Concentration	Dilution
AT8	Tau phosphorylated at Ser202/Thr205	Thermo Scientific, MA, USA	AB_223647	0.25 µg/ml	1:800
4G8	Amyloid-β_{17-24}	BioLegend, San Diego CA	AB_2564633	n.a.	1:1000
LB509	Lewy bodies purified from patients with DLB	Thermo Scientific, MA, USA	AB_2920932	60 µg/ml	1:500
KM51	Full length (monoclonal antibody), NCL-ASYN	Novocastra, Leica	AB_442103	n.a.	1:250
Anti-pTDP	TDP phosphorylated at Ser409/410	Cosmo Bio USA, CA, USA	AB_1961900	n.a.	1:6000
Anti-p62	P62-lck ligand$_{257-437}$	BD Biosciences, CA, USA	AB_398152	0.125 µg/ml	1:2000
